# Supplementary material for: Evaluation of Membrane Fouling Control for Brackish Water Treatment Using a Modified Polyamide Composite Nanofiltration Membrane
Source: Membranes (Basel). 2022 Dec 28;13(1):38. doi: 10.3390/membranes13010038 (PMC9866803; doi:10.3390/membranes13010038)
Supplement: Supplementary file 1 [file membranes-13-00038-s001.zip › membranes-2098912-supplementary.pdf]

membranes

Supplementary Materials

Evaluation of Membrane Fouling Control for Brackish Water Treatment Using a Modified Polyamide Composite Nanofiltration Membrane

Xuebai Guo [1,*], Cuixia Liu [2], Bin Feng [3] and Yuanfeng Hao [1]

[1] Department of Environmental Engineering, Henan Vocational College of Water Conservancy and Environment, Zhengzhou 450008, China
[2] School of Energy & Environment, Zhongyuan University of Technology, Zhengzhou 450007, China
[3] CCTEG Chongqing Engineering (Group) Co., Ltd., Chongqing 401331, China
* Correspondence: guoxuebai@126.com; Tel.: +86-186-0386-1013

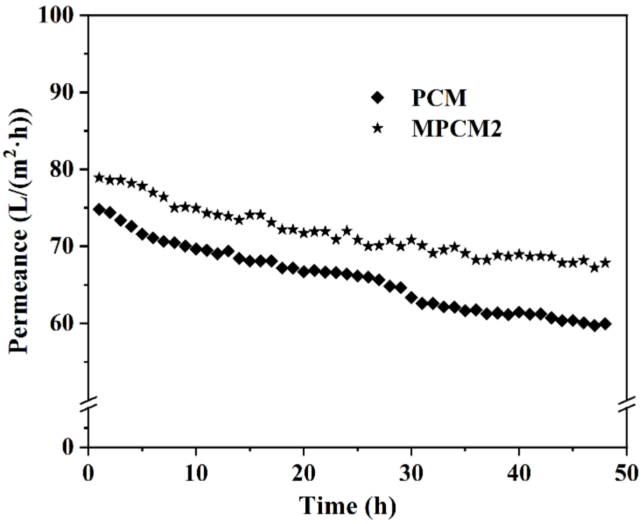

Figure S1. Membrane permeance of PCM and MPCM2 for the brackish water treatment.

Table S1. Feed quality of pretreated brackish water for nanofiltration experiments.

Species	Measured value	Species	Measured value (mg/L)
pH	8.1	Cl^- (mg/L)	373.98
Na^+ (mg/L)	350.71	SO_4^{2-} (mg/L)	746.82
K^+ (mg/L)	6.84	HCO_3^- (mg/L)	178.5
Ca^{2+} (mg/L)	96.85	NO_3^- (mg/L)	0.85
Mg^{2+} (mg/L)	107.53	F^- (mg/L)	0.74
Fe^{3+} (mg/L)	0.42	Turbidity (NTU)	1.3
COD (mg/L)	4.23	TDS (mg/L)	1890.35
Total hardness ($CaCO_3$, mg/L)	695.7		

Table S2. Formula for the four classical membrane fouling models.

Models	Equations
Complete Blocking	$J_0 - J = aV$
Standard Blocking	$\frac{1}{t} + b = \frac{J_0}{V}$
Intermediate Blocking	$\ln J_0 - \ln J = cV$
Cake Filtration	$\frac{1}{J} - \frac{1}{J_0} = dV$

where J_0 is the initial permeance ($m^3/[m^2 \cdot s]$), V is the filtrated volume (m^3), t is the filtration time (s), and a, b, c, and d are the constants.

Table S3. Volume ratio of permeate to feed flow (Y) at different operating conditions at the end of nanofiltration.

Pressure (MPa)	Y (%)	Temperature (°C)	Y (%)	Cross-flow velocity (cm/s)	Y (%)
0.3	26.92	20	41.15	3	36.53
0.5	43.18	25	43.18	4	43.18
0.7	51.88	30	44.87	7	44.54
0.9	60.88				

Table S4. Nanofiltration performance of pretreated brackish water in northwest China using MPCM2 at optimal operating condition.

Index	Feed concentration (mg/L)	Effluent concentration (mg/L)	Removal rates (%)	Stander value
pH	8.1	7.4	—	6.5~8.5
Na^+	350.71	183.61	47.65	200
K^+	6.84	5.43	20.61	—
Ca^{2+}	96.85	38.25	60.51	—
Mg^{2+}	107.53	55.32	48.55	—
Fe^{3+}	0.42	0.06	85.71	0.3
Cl^-	373.98	216.76	42.04	250
SO_4^{2-}	746.82	60.87	91.85	250
HCO_3^-	178.51	70.45	60.53	—
NO_3^-	0.85	0.42	50.59	10
F^-	0.74	0.41	44.59	1
COD	4.23	0.27	93.62	3
Total hardness	695.57	317.44	54.36	450
TDS	1890.35	642.65	66.00	1000
Turbidity	1.3 NTU	0.1 NTU	92.31	1 NTU

Table S5. Fitting parameters of the membrane permeance under the optimal operating conditions based on membrane fouling models.

Membrane fouling models	Fitting parameters	
	R^2	SSE
Complete blocking	0.90402	0.00816
Standard blocking	0.54311	5.45469E10
Intermediate blocking	0.92364	0.00650
Cake filtration	0.93947	0.00515
